# Impact of Antifibrotic Treatment on Postoperative Complications in Patients with Interstitial Lung Diseases Undergoing Lung Transplantation: A Systematic Review and Meta-Analysis

**DOI:** 10.3390/jcm12020655

**Published:** 2023-01-13

**Authors:** Pahnwat Taweesedt, Ploypin Lertjitbanjong, Dararat Eksombatchai, Prangthip Charoenpong, Teng Moua, Charat Thongprayoon, Supawit Tangpanithandee, Tananchai Petnak

**Affiliations:** 1 Division of Sleep Medicine, Department of Psychiatry and Behavioral Sciences, Stanford University School of Medicine, Palo Alto, CA 94305, USA; 2 Division of Pulmonary, Critical Care, and Sleep Medicine, Department of Medicine, University of Tennessee Health Science Center, Memphis, TN 38163, USA; 3 Division of Pulmonary and Pulmonary Critical Care Medicine, Department of Medicine, Faculty of Medicine Ramathibodi Hospital, Mahidol University, Bangkok 10400, Thailand; 4 Division of Pulmonary and Critical Care Medicine, Department of Internal Medicine, Louisiana State University Health Sciences Center at Shreveport, Shreveport, LA 71103, USA; 5 Division of Pulmonary and Critical Care Medicine, Mayo Clinic, Rochester, MN 55902, USA; 6 Division of Nephrology and Hypertension, Department of Medicine, Mayo Clinic, Rochester, MN 55902, USA; 7 Chakri Naruebodindra Medical Institute, Faculty of Medicine Ramathibodi Hospital, Mahidol University, Samut Prakan 10540, Thailand

**Keywords:** interstitial lung disease, lung transplantation, antifibrotic, pirfenidone, nintedanib, complication, postoperative

## Abstract

Antifibrotic treatment has been approved for reducing disease progression in fibrotic interstitial lung disease (ILD). As a result of increased bleeding risk, some experts suggest cessation of antifibrotics prior to lung transplantation (LT). However, extensive knowledge regarding the impact of antifibrotic treatment on postoperative complications remains unclear. We performed a comprehensive search of several databases from their inception through to 30 September 2021. Original studies were included in the final analysis if they compared postoperative complications, including surgical wound dehiscence, anastomosis complication, bleeding complications, and primary graft dysfunction, between those with and without antifibrotic treatment undergoing LT. Of 563 retrieved studies, 6 studies were included in the final analysis. A total of 543 ILD patients completing LT were included, with 161 patients continuing antifibrotic treatment up to the time of LT and 382 without prior treatment. Antifibrotic treatment was not significantly associated with surgical wound dehiscence (RR 1.05; 95% CI, 0.31–3.60; *I*^2^ = 0%), anastomotic complications (RR 0.88; 95% CI, 0.37–2.12; *I*^2^ = 31%), bleeding complications (RR 0.76; 95% CI, 0.33–1.76; *I*^2^ = 0%), or primary graft dysfunction (RR 0.87; 95% CI, 0.59–1.29; *I*^2^ = 0%). Finally, continuing antifibrotic treatment prior to LT was not significantly associated with decreased 1-year mortality (RR 0.80; 95% CI, 0.41–1.58; *I*^2^ = 0%). Our study suggests a similar risk of postoperative complications in ILD patients undergoing LT who received antifibrotic treatment compared to those not on antifibrotic therapy.

## 1. Introduction

Interstitial lung diseases (ILDs) or diffuse parenchymal lung diseases consist of a heterogeneous group of lung disorders involving varying degrees of interstitial inflammation and fibrosis. Progressive pulmonary fibrosis encompasses interstitial lung diseases with a progressive phenotype characterized by accelerated pulmonary function decline, worsening respiratory symptoms, and early mortality [1]. Male gender, older age, low baseline force vital capacity, and diffusion capacity for carbon monoxide are reported risk factors for ILD progression [2,3]. Pathogenesis of fibrosis in fibrotic ILD includes alveolar epithelial injury, unregulated repair, fibroblast proliferation, and increased extracellular matrix deposition resulting in decreased lung compliance [4]. Idiopathic pulmonary fibrosis (IPF) is the archetypical disease and most common form of ILD with progressive fibrosis. Other progressive fibrosing ILDs include the connective tissue disease-associated ILD, sarcoidosis, fibrotic hypersensitivity pneumonitis, idiopathic nonspecific interstitial pneumonia, cryptogenic organizing pneumonia, and unclassifiable ILD [5].

Antifibrotic treatment has been approved by the U.S. Food and Drug Administration for reducing pulmonary function decline. Both nintedanib and pirfenidone have been recommended in all patients with IPF based on high-quality randomized placebo-controlled trials [6,7,8]. However, only nintedanib has been studied and approved for non-IPF ILD with progressive fibrosis in a multicenter phase 3 trial [9]. Patients with progressive pulmonary fibrosis other than IPF may also benefit from pirfenidone based on recent phase 2 trials [10,11].

Nintedanib is an oral antifibrotic that inhibits intracellular tyrosine kinase leading to the blockage of fibroblast growth factor, platelet-derived growth factor (PDGF), and vascular endothelial growth factor (VEGF) receptors [12]. Pirfenidone demonstrates antifibrotic properties by attenuating transforming growth factor-β, a pro-fibrotic cytokine. Both medications decrease inflammation and hamper fibroblast migration, differentiation, and activation [13], and thus may theoretically delay wound healing and increase bleeding risk in postoperative patients. Furthermore, anastomotic complications are associated with high post-lung transplant morbidity [14]. As a result, some experts recommend discontinuing antifibrotic treatment before lung transplantation (LT) [15]. The premature stopping of antifibrotics may theoretically worsen the clinical course of those awaiting LT [16].

To date, LT remains the only definitive treatment for advanced-stage ILD. It has been shown to improve quality of life and survival [17,18]. Patients with ILD often receive antifibrotic therapy immediately prior to LT. Studies regarding the impact of antifibrotic treatment on postoperative outcomes in LT remain limited. Whether or not antifibrotic treatment should be discontinued before transplantation to allow for a wash-out period and avoid postoperative complications is unclear. Therefore, we conducted a systematic review and meta-analysis to assess the risk of antifibrotic treatment on post-transplant complications in ILD patients.

## 2. Materials and Methods

### 2.1. Search Strategy and Study Selection

The systematic review and meta-analysis were conducted and reported in accordance with the Preferred Reporting Items for Systematic Reviews and Meta-analyses (PRISMA) statement. A comprehensive search of several databases from their inception to 30 September 2021, in any language, was performed. The databases included Ovid MEDLINE(R) and Epub Ahead of Print, In-Process & Other Non-Indexed Citations, Daily, Ovid EMBASE, Ovid Cochrane Central Register of Controlled Trials, Ovid Cochrane Database of Systematic Reviews, and Scopus. The search strategy was designed and performed by an experienced librarian with input from the study’s principal investigator. Controlled vocabulary supplemented with keywords was used to search for studies of antifibrotic therapy on patients with interstitial lung diseases who underwent LT. The search strategy listing all terms and how they were combined is available in the Supplementary file.

Studies were included in the final meta-analysis if they directly compared postoperative complications after LT in patients with ILD receiving or not receiving antifibrotic treatment. Postoperative complications included surgical wound dehiscence, anastomosis complications, bleeding complications, primary graft dysfunction, and mortality. Primary graft dysfunction was evaluated within 72 h after the surgery. Studies in which full-text articles were not published or available for review were excluded. If studies were conducted with significantly overlapping datasets, those with larger patient numbers were included in the meta-analysis.

The titles and abstracts of all retrieved studies were independently reviewed by two investigators (P.T. and P.L.). Studies with disagreement on exclusion were included in the full-text review. Full-text articles included studies which were subsequently retrieved and independently reviewed by the same two investigators (P.T. and P.L.). Further disagreements on inclusion were adjudicated by a third reviewer (T.P.). The bibliographies of included studies were also reviewed to identify additional eligible studies. Percent agreement and κ statistics between the two reviewers were calculated to assess the reliability of the study selection.

### 2.2. Data Abstraction and Quality Assessment

Two investigators (P.T. and P.L.) independently abstracted data from eligible studies. The following variables were abstracted: author names, year of publication, participant characteristics (number, waitlist time, and lung allocation score at transplantation), antifibrotic subtype (nintedanib vs. pirfenidone), transplant procedures (bilateral vs. single LT and intraoperative extracorporeal life support), and postoperative complications, categorized as surgical wound dehiscence, anastomosis complications, bleeding complications, and primary graft dysfunction, between those with and without antifibrotic treatment.

Risk of bias for each study was independently evaluated by two reviewers (P.T. and T.P.) using the Newcastle–Ottawa scale [19]. Disagreements regarding data abstraction and risk of bias assessment were resolved through reviewer discussion.

### 2.3. Outcomes

The aim of the current systematic review and meta-analysis was to compare the risk of postoperative complications between ILD patients with and without antifibrotic treatment prior to LT, including surgical wound dehiscence, bronchial anastomosis complications, bleeding complications, primary graft dysfunction, and 1-year mortality after LT. The definition of primary graft dysfunction at 72 h after LT by the International Society for Heart and Lung Transplantation (ISHLT) was used [20]. Further analyses were conducted to evaluate the association of these outcomes and each antifibrotic treatment individually. Effect size was estimated as risk ratios (RRs) with a 95% confidence interval (CI) for all outcomes.

### 2.4. Statistical Analysis

A number of postoperative complications for patients with and without antifibrotic treatment prior to LT were abstracted from eligible studies. Pooled RR and 95% CI were estimated for each outcome using random-effect meta-analyses to account for differences in underlying ILD disease type and severity, and center experience for LT.

Statistical heterogeneity of effect size across included studies was assessed using the Cochran Q test and *I*^2^ statistic. A *p*-value of <0.10 on the Q test was considered significant. *I*^2^ values of 0–25% indicate no significant heterogeneity, 26–50% indicate low heterogeneity, 51–75% indicate moderate heterogeneity, and >75% indicate high heterogeneity [20]. Given the smaller number of included studies for each outcome, publication bias assessment and subgroup analysis were not performed. All statistical analysis was performed using RevMan 5.4 (Cochrane Collaboration, Oxford, UK).

## 3. Results

**Figure 1:**
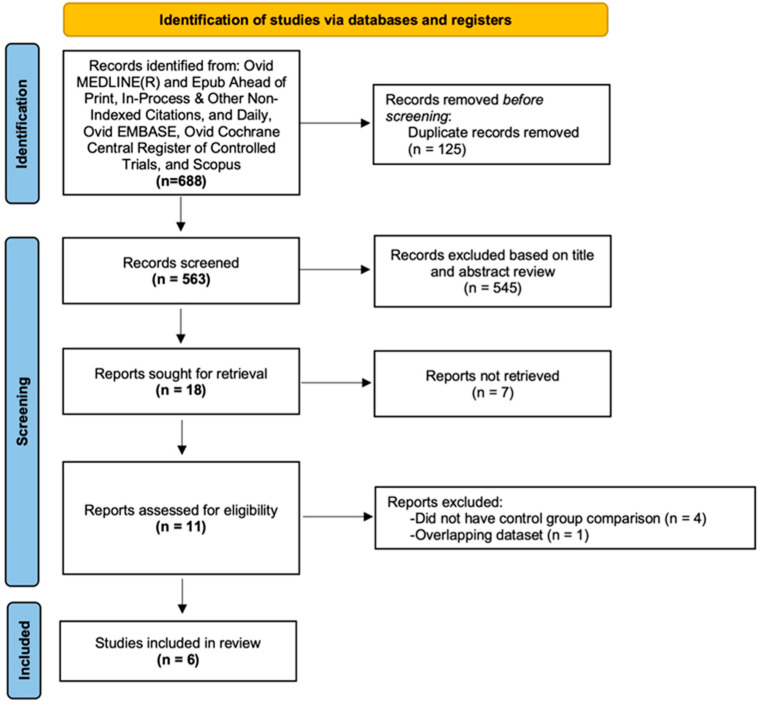
Study selection.

A total of 563 articles were identified in our search, of which 545 were excluded based on title and abstract review. Of the 11 studies undergoing full-text review, six were included in the final meta-analysis, as shown in Figure 1. The κ statistic and percent agreement for study selection were 0.81 and 90.9%, respectively.

Characteristics of included studies are presented in Table 1. A total of 161 ILD patients remained in antifibrotic therapy up to the time of LT and 382 not in therapy were included. Of the six included studies, one was a case series [12], while the remaining studies were observational studies [21,22,23,24,25]. The mean duration of antifibrotic treatment was reported in four studies, ranging from 419 to 1356 days. Waitlist time and lung allocation score were reported in most studies. All included studies were considered to be at risk of bias using the Newcastle–Ottawa scale (Table 2).

### 3.1. Postoperative Complications

**Figure 2:**
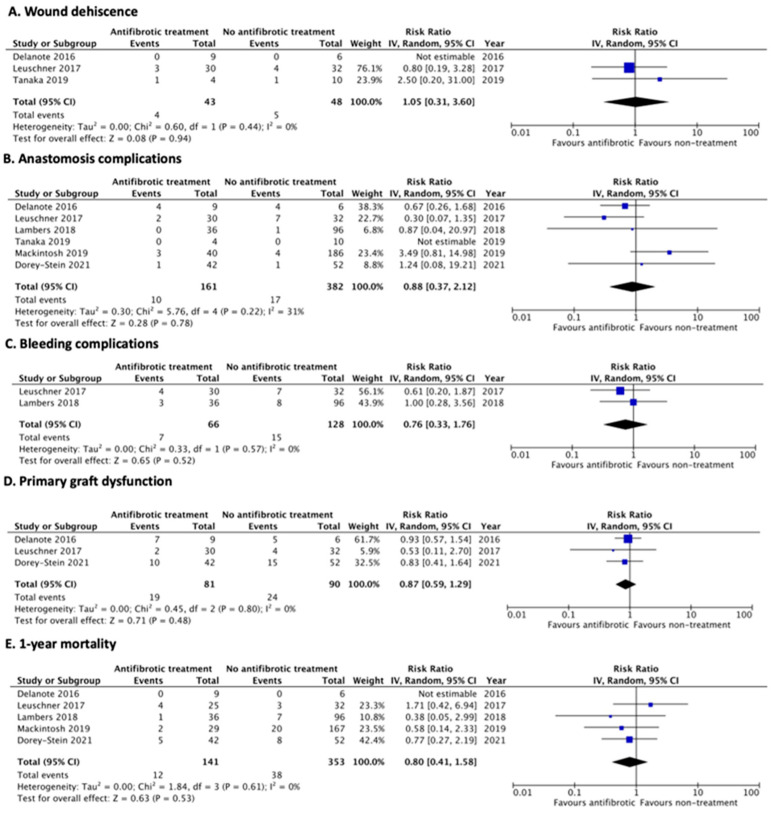
Forest plot of pooled risk ratio for all antifibrotic treatments [12,21,22,23,24,25]. (**A**) surgical wound dehiscence, (**B**) anastomosis complications, (**C**) bleeding complications, (**D**) primary graft dysfunction at 72 h after transplantation, and (**E**) 1-year mortality after LT.

Three studies were included in the analysis of surgical wound dehiscence, including 43 patients with antifibrotic treatment prior to LT and 48 without the treatment. Antifibrotic treatment was not significantly associated with surgical wound dehiscence, with an RR of 1.05 (95% CI, 0.31–3.60; *I*^2^ = 0%) (Figure 2A). For anastomosis complications, 161 patients receiving antifibrotics and 382 patients not receiving them were included from six studies. Antifibrotic treatment was not significantly associated with anastomosis complications, with an RR of 0.88 (95% CI, 0.37–2.12; *I*^2^ = 31%) (Figure 2B). Regarding bleeding complications, pooled analysis from two studies demonstrated that antifibrotic use was not significantly associated with bleeding complications, with an RR of 0.76 (95% CI, 0.33–1.76; *I*^2^ = 0%) (Figure 2C). Furthermore, antifibrotic therapy was not significantly associated with primary graft dysfunction at 72 h after transplantation, with an RR of 0.87 (95% CI, 0.59–1.29; *I*^2^ = 0%). Finally, continuing antifibrotic treatment prior to LT was not significantly associated with decreased 1-year mortality after LT, with an RR of 0.80 (95% CI, 0.41–1.58; *I*^2^ = 0%).

### 3.2. Impact of Each Antifibrotic Treatment on Postoperative Complications

**Figure 3:**
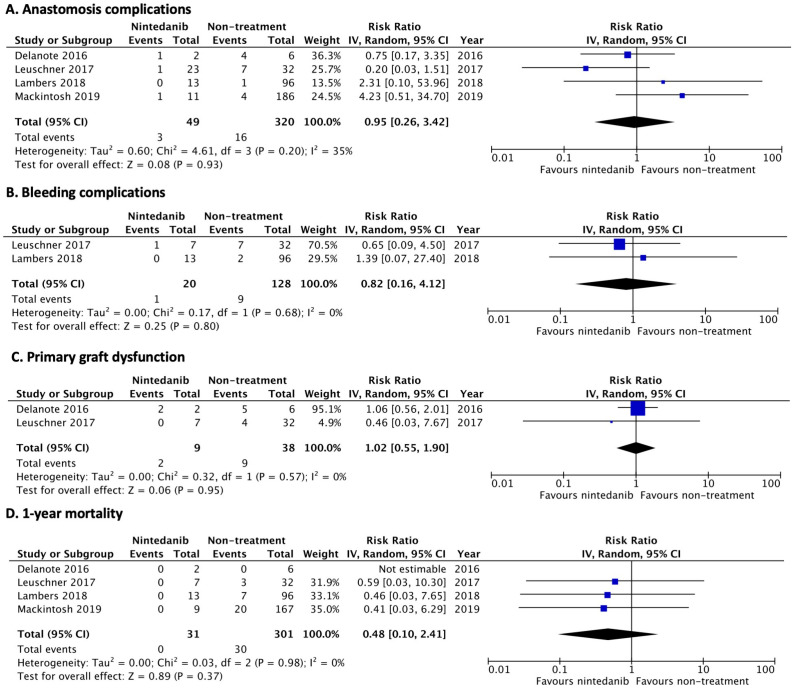
Forest plot of pooled risk ratio for nintedanib [12,21,22,23]. (**A**) anastomosis complications, (**B**) bleeding complications, (**C**) primary graft dysfunction at 72 h after transplantation, and (**D**) 1-year mortality after LT.

When analysis was separately performed for individual antifibrotics, continuing nintedanib prior to LT was not associated with anastomosis complications (RR 0.95; 95% CI, 0.26–3.42; *I*^2^ = 35%), major bleeding (RR 0.82; 95% CI, 0.16–4.12; *I*^2^ = 0%), or primary graft dysfunction (RR 1.02; 95% CI, 0.55–1.90; *I*^2^ = 0%). In addition, treatment with nintedanib prior to LT was not significantly associated with decreased 1-year mortality after LT (RR 0.48; 95% CI, 0.10–2.41; *I*^2^ = 0%) (Figure 3). Two studies reported wound dehiscence complications among patients treated with nintedanib [12,21]. Of nine patients treated with nintedanib prior to LT, no patients developed wound dehiscence, while four of 38 (11%) patients who did not receive antifibrotics had wound dehiscence.

**Figure 4:**
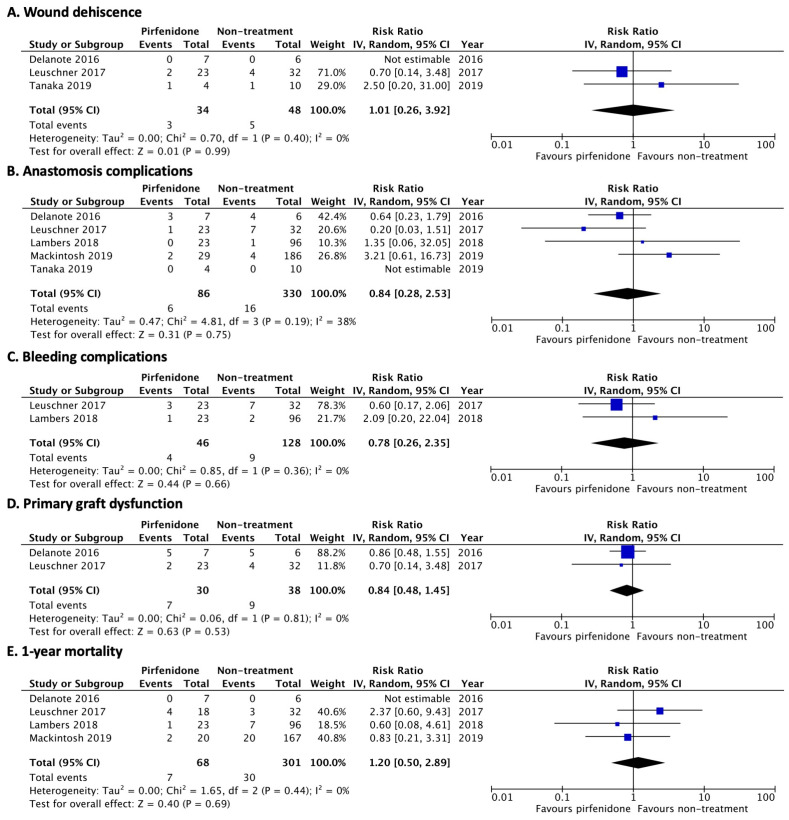
Forest plot of pooled risk ratio for pirfenidone [12,21,22,23,24]. (**A**) surgical wound dehiscence, (**B**) anastomosis complications, (**C**) bleeding complications, (**D**) primary graft dysfunction at 72 h after transplantation, and (**E**) 1-year mortality after LT.

Similarly, continuing pirfenidone was not associated with wound dehiscence (RR 1.01; 95% CI, 0.26–3.92; *I*^2^ = 0%), anastomosis complications (RR 0.84; 95% CI, 0.28–2.53; *I*^2^ = 38%), major bleeding (RR 0.78; 95% CI, 0.26–2.35; *I*^2^ = 0%), any grade of primary graft dysfunction (RR 0.84; 95% CI, 0.48–1.45; *I*^2^ = 0%), or 1-year mortality after LT (RR 1.20; 95% CI, 0.50–2.89; *I*^2^ = 0%) (Figure 4).

Specifically, grade-three primary graft dysfunction was reported in two studies. There were grade-three primary graft dysfunctions in 2 of 23 patients in the pirfenidone group, none in the nintedanib group (0 of 7), 4 of 32 patients in the control group from the study by Leuschner et al., 1 of 7 patients in the pirfenidone group, 1 of 2 patients in the nintedanib group and 1 of 6 patients in the control group from the study by Delanote and colleagues. Combining the results from these two studies, the rates of grade-three primary graft dysfunction were 10% in the pirfenidone group, 11% in the nintedanib group, and 13% in the control group [12,22].

## 4. Discussion

LT is known to decrease mortality in pulmonary fibrosis. Nonetheless, the long-term survival rate of patients who undergo transplantation remains limited compared to other solid organ transplants [26]. This may be related to perioperative surgical and medical complications among post-LT patients. As indications for antifibrotics in pulmonary fibrosis expand, post-transplant outcomes in patients with ILD who are treated with antifibrotics have been studied, including anastomosis complications, surgical wound dehiscence, primary graft dysfunction, bleeding complications, blood transfusions, acute cellular rejection, chronic lung allograft dysfunction, mechanical ventilation duration, and early and late mortality [27].

In this systematic review and meta-analysis, we focused on four main detrimental postoperative complications: surgical wound dehiscence, anastomosis complications, bleeding complications, and primary graft dysfunction. These events have been associated with higher graft failure and posttransplant morbidity, and mortality [28].

Our study showed that antifibrotic treatment did not have significant association with surgical wound dehiscence or risk of anastomosis complications. Since nintedanib and pirfenidone worked through different mechanisms, we performed the review and analyzed the outcome for each antifibrotic treatment separately. These results remain nonsignificant despite the assessment of each antifibrotic treatment individually. Nevertheless, the number of studies is limited. Anastomotic airway complications can be related to the impairment of blood flow and typically occur during the first month after transplantation [29]. Even though antifibrotics are thought to delay wound healing and increase the risk of surgical wound dehiscence and anastomosis complications, the lack of this effect may be due to the short half-life of antifibrotics, 10–15 h in nintedanib and 2.4 h in pirfenidone [30,31]. Accordingly, nintedanib might have had a greater effect on these peri-transplant complications than pirfenidone [23]. More participants in our meta-analysis were on pirfenidone than nintedanib; a prospective study with a larger population of those on nintedanib is warranted.

Primary graft dysfunction is a severe acute complication of LT due to non-immune mediated ischemic reperfusion injury. It presents as bilateral lung infiltrates and hypoxemia (PaO_2_/FiO_2_ < 300) within 72 postoperative hours after excluding other causes such as infection or cardiogenic pulmonary edema. Primary graft dysfunction can give rise to early postoperative mortality and chronic lung allograft dysfunction [28]. The anti-inflammatory profiles of antifibrotic therapies are thought to alleviate ischemia reperfusion injury, as seen in pirfenidone with rat models [32]. This promising finding suggests the potential benefit of antifibrotics in transplant patients. Our study failed to present the advantage of pre-transplant antifibrotics in decreasing the risk of primary graft dysfunction.

Nintedanib’s hindrance of VEGF and PDGF receptors may interfere with the revascularization process and increase bleeding risk [12]. According to this reason, some experts recommend discontinuing nintedanib prior to LT. Our results did not show increased bleeding risk with the use of antifibrotics. In addition, our pooled analysis demonstrates no increased risk for bleeding complications in patients continuing nintedanib prior to LT. Again, the number of included studies was limited. Further studies with a larger population of patients undergoing nintedanib treatment are required.

Our study represents the first meta-analysis of antifibrotic impact on LT complications in pulmonary fibrosis. Heterogeneity in our study is low in most pooled analyses. Our results should be carefully interpreted given necessary limitations. First, the number of included studies was small (three studies for surgical wound dehiscence, six for anastomosis complications, two for bleeding complications, and three for primary graft dysfunction). All included studies were retrospective cohorts and case series with smaller numbers. Most studies were also single center reports. Apart from the primary graft dysfunction whereby the studies unanimously used the definition from the ISHLT, most of the other outcomes were not precisely defined. There was only one study from Mackintosh et al. that reported the detail of the bronchial anastomotic dehiscence definition [24].

Reporting of the baseline characteristics of participants was not standardized with the majority of participants included in our meta-analysis on pirfenidone rather than nintedanib. There was one study that reported the number of participants involved in steroid use in the non-antifibrotic group which would affect postoperative complications [23]. Unlike primary graft dysfunction, the post operative follow-up duration for the other outcomes that we analyzed was not clearly demonstrated. Only the study from Lambers et al. reported all of the outcomes within four weeks after surgery or until hospital discharge [23]. Furthermore, long-term outcomes are unable to be evaluated since the included studies focused on the postoperative period. Other post-transplant outcomes such as acute cellular rejection, bronchiolitis obliterans syndrome, restrictive allograft syndrome, chronic allograft dysfunction, and mortality were not able to be analyzed due to the lack of consistently reported data. Moreover, the type of LT (single vs. bilateral LT) can have an impact on postoperative outcome. Nonetheless, the outcome of patients with and without antifibrotics in each type of LT was not enough to conduct subgroup analysis of all of the studies. In the study by Leuschner, bilateral LT showed no survival benefit over single LT in the entire cohort as well as in a subgroup of pirfenidone vs. the control. In this study, nintendanib outcome was not performed because only one patient on nintedanib underwent bilateral LT [22].

Finally, the perioperative extracorporeal membrane oxygenation (ECMO) use is one of the crucial risk factors associated with postoperative complications. The perioperative ECMO rate in our included studies ranges from 5–65%. There was limited data regarding the effect of perioperative ECMO use on postoperative outcomes among patients with and without antifibrotic treatment. However, postoperative ECMO use varies among the LT center, either with regard to systematic use in all postoperative cases or use in selected cases.

In the current clinical practice guideline from the ISHLT, antifibrotics may be continued in patients with ILD on the transplant waitlist until the time of LT [33]. Further studies with prospective designs and larger numbers of participants are needed to better investigate the risks and benefits of perioperative antifibrotic utilization in those with advanced stage pulmonary fibrosis awaiting LT, in order to establish substantial evidence for practice guidelines.

## 5. Conclusions

In summary, our analysis failed to demonstrate significant differences in postoperative complications in those receiving or not receiving pre-transplant antifibrotic therapy, including anastomosis complications, surgical wound dehiscence, primary graft dysfunction, and bleeding complications. This suggests that antifibrotics may be continued until the time of LT with minimal postoperative effect.

## Figures and Tables

**Table 1:** Baseline characteristics of included studies.

Studies	Number of Participants		Duration of Antifibrotic Treatment (Days)	LAS		Waitlist Time		Bilateral Transplantation		Intra-Operative ECMO
	Antifibrotic	No Antifibrotic		Antifibrotic	No Antifibrotic	Antifibrotic	No Antifibrotic	Antifibrotic	No Antifibrotic	
Delanote [12]	7 ^¶^ 2 ^†^	6	419 ± 315	32.4 ± 2.8 ^¶^ 31.5 ± 0.7 ^†^	31.5 ± 3.4	155 (40–299)	203 (88–275)	6 ^¶^ 2 ^†^	5	1(7%) ^¶^
Leuschner [21]	23 ^¶^ 7 ^†^	32	591 ± 402	52.1 ± 15.5 ^¶^ 50.3 ± 18.8 ^†^	54.5 ± 16.7	122±369	NA	13 ^¶^ 1 ^†^	20	30 (48%)
Lambers [22]	23 ^¶^ 13 ^†^	96	NA	38 (26–50) ^¶^ 37 (33–40) ^†^	39 (34–45) ^‡^ 37 (33–40) ^§^	NA	NA	23 ^¶^ 13 ^†^	96	74 (56%)
Mackintosh [23]	29 ^¶^ 11 ^†^	186	NA	NA	NA	53 (6–655) *	77 (0–1195) *	34	159	146 (65%)
Tanaka [24]	4 ^¶^	10	1356 (558–2004)	35.2 (34–39) ^¶^	42.5 (33–56)	438 (241–910)	389 (8–1366)	2 ^¶^	5	NA
Dorey-Stein [25]	28 ^¶^ 14 ^†^	52	579 ± 597	45.1 ± 14.1	53.9 ± 20.4	166	150	16	16	5 (5%)

Data presented as mean ± SD, median (interquartile range), or frequency (%). LAS = lung allocation score, ECMO = extracorporeal membrane oxygenation. * median (range). ^¶^ pirfenidone, ^†^ nintedanib, ^‡^ corticosteroid treatment, ^§^ no treatment.

**Table 2:** Risk of bias assessment for cohort study using Newcastle–Ottawa Scale.

Studies	Selection	Comparability	Outcome
Delanote et al. [12]	★★★	-	★★★
Leuschner et al. [21]	★★★★	-	★★
Lambers et al. [22]	★★★★	-	★★
Mackintosh et al. [23]	★★★★	-	★★
Tanaka et al. [24]	★★★★	-	★★
Dorey-Stein et al. [25]	★★★★	-	★★★

Each star (★) represents one score for the individual domian of the Newcastle-Ottawa scale.

## Data Availability

The data presented in this study are available upon request from the corresponding author.

## Supplementary Materials

The following supporting information can be downloaded at https://www.mdpi.com/article/10.3390/jcm12020655/s1.
