# Supplementary material for: Impact of Antifibrotic Treatment on Postoperative Complications in Patients with Interstitial Lung Diseases Undergoing Lung Transplantation: A Systematic Review and Meta-Analysis
Source: J Clin Med. 2023 Jan 13;12(2):655. doi: 10.3390/jcm12020655 (PMC9865259; doi:10.3390/jcm12020655)
Supplement: Supplementary file 1 [file jcm-12-00655-s001.zip › jcm-2042815-supplementary.pdf]

Actual search strategy

Ovid

Database(s): EBM Reviews - Cochrane Central Register of Controlled Trials July 2021, EBM Reviews - Cochrane Database of Systematic Reviews 2005 to August 18, 2021, Embase 1974 to 2021 August 20 , Ovid MEDLINE(R) and Epub Ahead of Print, In-Process, In-Data-Review & Other Non-Indexed Citations and Daily 1946 to August 20, 2021
Search Strategy:

#	Searches	Results
1	exp Lung Transplantation/	54890
2	((lung or pulmonary) adj3 (transplant* or graft* or allotransplant* or "allo-transplant*" or homotransplant* or "homo-transplant*" or retransplant* or "re-transplant*" or autotransplant* or "auto-transplant*" or allograft* or "allo-graft*" or homograft* or "homo-graft*" or "cadaver lung*" or heterotransplant* or "hetero-transplant*")).ti,ab,kw.	62471
3	1 or 2	77489
4	exp Lung Diseases, Interstitial/	146044
5	exp Idiopathic Pulmonary Fibrosis/	33971
6	(Anthracosilicosis or Anthracosis or "anti-gbm disease*" or "anti-glomerular basement membrane disease*" or Asbestosis or bagassosis or Berylliosis or "Bird Fancier Lung" or "Bird Fanciers Lung" or Byssinosis or "Caplan Syndrome*" or "Caplans Syndrome*" or "Cryptogenic Organizing Pneumonia*" or "diffuse parenchymal lung disease*" or "Eosinophilic Granuloma*" or "extrinsic allergic alveolitides" or "extrinsic allergic alveolitis" or "Farmer Lung" or "Farmers Lung" or "generalized histiocytoses" or "generalized histiocytosis" or "goodpasture syndrome*" or "goodpastures syndrome*" or "granulomatosis with polyangiitides" or "granulomatosis with polyangiitis" or "Hamman-Rich Syndrome*" or "hand-schueller-christian disease*" or "hand-schueller-christian syndrome*" or "hand-schuller-christian disease*" or "hand-schuller-christian syndrome*" or "hashimoto-pritzger disease*" or "histiocytosis-x" or "hypersensitivity pneumonitides" or "hypersensitivity pneumonitis" or "idiopathic interstitial pneumonia*" or "idiopathic pulmonary fibros*" or "interstitial lung disease*" or "interstitial pneumonia*" or "interstitial pneumonitides" or "interstitial pneumonitis" or "interstitial pulmonary disease*" or IPF or "langerhans-cell granulomatoses" or "langerhans-cell granulomatosis" or "langerhans-cell histiocytoses" or "langerhans-cell histiocytosis" or "letterer-siwe disease*" or "lung purpura with nephritis" or "non-lipid reticuloendothelioses" or "non-lipid reticuloendotheliosis" or pneumoconioses or pneumoconiosis or "pulmonary histiocytosis x" or "pulmonary langerhans cell granulomatosis" or "pulmonary sarcoidoses" or "pulmonary sarcoidosis" or "radiation fibros*" or "radiation pneumonia*" or "radiation pneumonitis" or "schueller-christian disease*" or Siderosis or Silicosis or Silicotuberculosis or "Silo Filler Disease*" or "Silo Fillers Disease*" or "systemic aleukemic reticuloendothelioses" or "systemic aleukemic reticuloendotheliosis" or	174436

	Trichosporonosis or "type 2 histiocytoses" or "type 2 histiocytosis" or "wegener granulomatosis" or "wegeners granulomatosis").ti,ab,kw.	
7	4 or 5 or 6	227162
8	3 and 7	7278
9	exp antifibrotic agent/	20343
10	exp nintedanib/	3776
11	exp pirfenidone/	4000
12	(antifibrotic* or "anti-fibrotic*" or nintedanib or pirfenidone).ti,ab,kw.	28183
13	9 or 10 or 11 or 12	49004
14	8 and 13	802
15	(case* adj3 report*).mp,pt.	5276292
16	14 not 15	716
17	limit 16 to (editorial or erratum or note or addresses or autobiography or bibliography or biography or blogs or comment or dictionary or directory or interactive tutorial or interview or lectures or legal cases or legislation or news or newspaper article or overall or patient education handout or periodical index or portraits or published erratum or video-audio media or webcasts) [Limit not valid in CCTR,CDSR,Embase,Ovid MEDLINE(R),Ovid MEDLINE(R) Daily Update,Ovid MEDLINE(R) PubMed not MEDLINE,Ovid MEDLINE(R) In-Process,Ovid MEDLINE(R) Publisher; records were retained]	28
18	16 not 17	688
19	remove duplicates from 18	563

Scopus

1 TITLE-ABS-KEY(((lung or pulmonary) W/3 (transplant* or graft* or allotransplant* or "allo-transplant*" or homotransplant* or "homo-transplant*" or retransplant* or "re-transplant*" or autotransplant* or "auto-transplant*" or allograft* or "allo-graft*" or homograft* or "homo-graft*" or "cadaver lung*" or heterotransplant* or "hetero-transplant*")))

2 TITLE-ABS-KEY(Anthracosilicosis OR Anthracosis OR "anti-gbm disease*" OR "anti-glomerular basement membrane disease*" OR Asbestosis OR bagassosis OR Berylliosis OR "Bird Fancier Lung" OR "Bird Fanciers Lung" OR Byssinosis OR "Caplan Syndrome*" OR "Caplans Syndrome*" OR "Cryptogenic Organizing Pneumonia*" OR "diffuse parenchymal lung disease*" OR "Eosinophilic Granuloma*" OR "extrinsic allergic alveolitides" OR "extrinsic allergic alveolitis" OR "Farmer Lung" OR "Farmers Lung" OR "generalized histiocytoses" OR "generalized histiocytosis" OR "goodpasture syndrome*" OR "goodpastures syndrome*" OR "granulomatosis with polyangiitides" OR "granulomatosis with polyangiitis" OR "Hamman-Rich Syndrome*" OR "hand-schueller-christian disease*" OR "hand-schueller-christian syndrome*" OR "hand-schuller-christian disease*" OR "hand-schuller-christian syndrome*" OR "hashimoto-pritzger disease*" OR "histiocytosis-x" OR "hypersensitivity pneumonitides" OR "hypersensitivity pneumonitis" OR "idiopathic interstitial pneumonia*" OR "idiopathic pulmonary fibros*" OR "interstitial lung disease*" OR "interstitial pneumonia*" OR "interstitial pneumonitides" OR "interstitial pneumonitis" OR "interstitial pulmonary disease*" OR IPF OR "langerhans-cell granulomatoses" OR "langerhans-cell granulomatosis" OR "langerhans-cell histiocytoses" OR "langerhans-cell histiocytosis" OR "letterer-siwe disease*" OR "lung purpura with nephritis" OR "non-lipid reticuloendothelioses" OR "non-lipid reticuloendotheliosis" OR pneumoconioses OR pneumoconiosis OR "pulmonary histiocytosis x" OR "pulmonary langerhans cell granulomatosis" OR "pulmonary sarcoidoses" OR "pulmonary sarcoidosis" OR "radiation fibros*" OR "radiation pneumonia*" OR "radiation pneumonitis" OR "schueller-christian disease*" OR Siderosis OR Silicosis OR Silicotuberculosis OR "Silo Filler Disease*" OR "Silo Fillers Disease*" OR "systemic aleukemic reticuloendothelioses" OR "systemic aleukemic reticuloendotheliosis" OR Trichosporonosis OR "type 2 histiocytoses" OR "type 2 histiocytosis" OR "wegener granulomatosis" OR "wegeners granulomatosis")

3 TITLE-ABS-KEY(antifibrotic* OR "anti-fibrotic*" OR nintedanib OR pirfenidone)

4 1 and 2 and 3

5 TITLE-ABS-KEY(case* W/3 report*)

6 4 and not 5

7 DOCTYPE(ed) OR DOCTYPE(bk) OR DOCTYPE(er) OR DOCTYPE(no) OR DOCTYPE(sh)

8 6 and not 7

9 INDEX(embase) OR INDEX(medline) OR PMID(0* OR 1* OR 2* OR 3* OR 4* OR 5* OR 6* OR 7* OR 8* OR 9*)

10 8 and not 9
